# Effect of dietary tannins on the performance, lymphoid organ weight, and amino acid ileal digestibility of broiler chickens: A meta-analysis

**DOI:** 10.14202/vetworld.2021.1405-1411

**Published:** 2021-06-01

**Authors:** Cecep Hidayat, Agung Irawan, Anuraga Jayanegara, Muhammad Miftakhus Sholikin, Tri Rachmanto Prihambodo, Yulianri Rizki Yanza, Elizabeth Wina, Sadarman Sadarman, Rantan Krisnan, Isbandi Isbandi

**Affiliations:** 1 Indonesian Research Institute For Animal Production, Ciawi Bogor 16720, Indonesia; 2 Animal Feed and Nutrition Modelling Research Group, Faculty of Animal Science, IPB University, Bogor 16680, Indonesia; 3 Vocational School, Universitas Sebelas Maret, Surakarta 57126, Indonesia; 4 Department of Nutrition and Feed Technology, Faculty of Animal Science, IPB University, Bogor 16680, Indonesia; 5 Graduate Study Program of Nutrition and Feed Science, Faculty of Animal Science, IPB University, Bogor 16680, Indonesia; 6 Department of Animal Nutrition, Faculty of Veterinary Medicine and Animal Science, Poznan University of Life Sciences, Poznań 60-637, Poland; 7 Department of Animal Science, Sultan Syarif Kasim State Islamic University, Pekanbaru 28293, Indonesia; 8 Center for Livestock Studies and Development, Pahlawan Tuanku Tambusai University, Bangkinang 28412, Indonesia

**Keywords:** amino acid ileal digestibility, broiler chicken, meta-analysis, tannin

## Abstract

**Background and Aim:**

Tannins are functional secondary metabolites that may provide benefits to ruminants. However, to date, their effects on broiler chickens remain inconclusive. This study aimed to evaluate the effectiveness of dietary tannin levels on the performance, body organs, and amino acid (AA) digestibility of broiler chickens using a meta-analysis.

**Materials and Methods:**

After verification and evaluation, a total of 22 articles were included in the present study. All data regarding dietary tannin dosages, performance, digestibility, and gastrointestinal physiology of broiler chickens were tabulated into a database. The database data were then statistically analyzed using mixed models, with tannin dose as a fixed effect and study as a random effect.

**Results:**

High levels of dietary tannins negatively affected the average daily gain and average daily feed intake of broiler chickens according to linear patterns (p<0.001). In addition, dietary tannins decreased drumstick and liver weights, as well as bursa of Fabricius and spleen weight (p<0.05). Meanwhile, other carcass traits (i.e., thigh, wings, and body fat) were not influenced by dietary tannins. Regarding AA digestibility, high dietary tannin concentrations induced negative responses on isoleucine, leucine, and methionine digestibility (p<0.05).

**Conclusion:**

Dietary tannins appear to have a negative effect on broiler performance, lymphoid organ weight, and AA ileal digestibility. Hence, the addition of tannins to broiler diets is not recommended.

## Introduction

In the past decade, there has been intense discussion regarding an increased demand for functional food derived from broiler chickens. Since a global ban on the use of antibiotics as growth promoters, there has been a driving effort to utilize natural substances as feed additives in broiler production [1,2]. The use of growth promoters causes resistance to microbes, such that they are no longer effective in killing microorganisms of similar species [3]. Several alternatives have been evaluated by both farmers and researchers, with mixed results. Plant bioactive substances and their constituents are known to have medicinal abilities [4,5]. Since 1995, the use of plants as an alternative treatment to improve the health of animals has been referred to as naturopathy; tannins are a potentially useful compound since they have been widely recognized for their positive effects in some animal species, particularly ruminants.

Despite their antibacterial [6], anti-inflammatory [7], and antiviral [8] effects, plants containing tannins have not been widely used as feed additives in broiler chickens. Tannins are contained in several feed ingredients commonly used in broiler diets, such as sorghum and barley. Tannins are produced by green plants in different levels and qualities. In broiler chickens, the inclusion of up to 3% dietary tannins can improve gut health and digestive performance [9-11]. Unlike in ruminant animals, the mode of action of tannins in broiler chickens has not been fully characterized [9]. The previous studies have found inconsistent results: Some studies have demonstrated a positive effect of the addition of tannins in terms of improved performance, digestibility [12], and organ health [13], whereas other studies have reported negative effects due to their addition.

Because of the variability of results across different experiments, the data from various studies must be summarized to reach a robust conclusion concerning the effects of tannins on broilers. Therefore, this study aimed to evaluate the effectiveness of dietary tannin levels on the performance, organweight, and digestibility of broiler chickens using a meta-analysis methodology.

## Materials and Methods

### Ethical approval

Ethical approval is not necessary for this type of study.

### Database development

All the data used in the present study were collected from published articles and recorded in a database. Published articles were browsed using multiple search engines for scientific papers, such as Google Scholar, Scopus, Web of Science, PubMed, and Mendeley, using the keywords “tannin” and “broiler.” Approximately 187 articles were retrieved that outlined studies of tanning supplementation for broilers, yet only 120 articles of these articles showed potential for inclusion on the basis of their title and abstract. The parameters included were (1) broiler chicken performance: Average daily gain (ADG), feed intake (FI), feed conversion ratio (FCR), mortality, and ­carcass cut weight and (2) broiler chicken gastrointestinal physiology and digestibility: The weight of lymphoid organs, tibia minerals, nutrient digestibility, intestinal histomorphometry, and malondialdehyde concentration.

After careful evaluation, 22 articles were selected for inclusion in the database (Table-1) [12,14-34]. The selected articles confirmed that several forms of tannins have been used to supplement broiler diets, such as total tannins, condensed tannin, hydrolysable tannin, and tannic acid. Broiler chickens were mostly fed with a corn-soybean meal-based diet. Tannins were supplemented at a 0–30 g/kg of diet. Tannin supplementation dosages and their typical source were compiled in the database. Performance parameters such as ADG and FI were also compiled in the database, expressed as g/bird/day unit. Mortality and digestibility were expressed as percentages (%). If any data used a different measurement unit (e.g., %v/v or %w/v), these data were transformed into the above measurement units for consistency. Once all data for dietary tannin dosages, performance, digestibility, and gastrointestinal physiology of broiler chickens were established, the database was statistically analyzed.

**Table-1:** Studies included in the meta-analysis

No.	Reference	Period (d)	Tannin source	Tannin form	Dose (g/kg)
1	Ramah *et al*. [14]	35 sd 42	Commercial tannic acid	Tannic acid	0 sd 30
2	Mannelli *et al*. [15]	1 sd 35	Extracted from the wood of *C*. *sativa*	Hydrolysable tannin	0 sd 2.7
3	Manyelo *et al*. [16]	1 sd 21	*S. bicolor*	Condensed tannin	0 sd 1.2
4	Saleh *et al*. [17]	15 sd 27	Sorghum	Unspecified	0 sd 3.1
5	Bayerle *et al*. [18]	21 sd 42	Black wattle tannin (*Acacia mearnsii*)	Tannic acid	0 sd 0.9
6	Tomaszewska *et al*. [19]	1 sd 21	Faba bean (FB) seeds (*V. faba* L. minor)	Unspecified	0 sd 1.6
7	Abdulla *et al*. [20]	13 sd 21	Field beans (*V. faba* L. var. minor)	Condensed tannin	0 sd 1.86
8	Bayerle *et al*. [21]	1 sd 21	Wattle tannin (tannin acid 72%)	Tannic acid	0 sd 0.9
9	Xu *et al*. [22]	1 sd 21	*S. bicolor* L. Moench	Unspecified	0 sd 2.54
10	Abdulla *et al*. [23]	7 sd 16	Field beans (*V. faba* L. var. minor)	Unspecified	0 sd 25.4
11	Batonon-Alavo *et al*. [24]	8 sd 15	Sorghum, cottonseed meal, and millet	Condensed tannin	0 sd 0.89
12	Hu *et al*. [25]	1 d 42	Fermented rapeseed meal	Unspecified	0 sd 1.4
13	Starčević *et al*. [26]	1 sd 35	Commercial tannic acid	Tannic acid	0 sd 5
14	Hejdysz *et al*. [27]	1 sd 21	Pea (*P. sativum*)	Unspecified	0 sd 0.030
15	Riasi *et al*. [28]	1 sd 42	Pea seed (*Lathyrus sativus*)	Unspecified	0 sd 0.82
16	Rezar and Salobir [29]	28 sd 33	Sweet chestnut (*C. sativa* Mill.) wood extract	Unspecified	0 sd 1.5
17	Tandiang *et al*. [12]	1 sd 21	Sorghum	Condensed tannin	0 sd 1.32
18	Konieczka *et al*. [30]	8 sd 15	Peas (*P. sativum* L.)	Unspecified	0 sd 0.76
19	Torres *et al*. [31]	1 sd 42	Sorghum	Unspecified	0 sd 2.87
20	Sasipriya and Siddhuraju [32]	1 sd 21	*Entada scandens*, *Canavalia gladiata,* and *Canavalia ensiformis*	Unspecified	0 sd 0.0525
21	Teteh *et al*. [33]	1 sd 28	*Moringa oleifera*	Unspecified	0 sd 0.40
22	Brus *et al*. [34]	28 sd 42	Chestnut tannins	Hydrolysable tannin	0 sd 0.2

*C. sativa=Castanea sativa, P. sativum=Pisum sativum, S. bicolor=Sorghum bicolor, V. faba=Vicia faba*

### Statistical analysis

Data with compatible measurement units were processed for statistical analysis using a mixed model procedure for meta-analysis [35,36,37]. The analysis was run with SAS version 9.1 using the PROC MIXED procedure. Tannin supplementation level was set as a fixed effect, whereas the study was set as a random effect; thus, the analysis included a RANDOM statement. Statistical significance was set at p<0.05, whereas a trend was set p=0.05-0.10.

The tannins levels were treated as continuous predictor where the response variables were regressed using the following mathematical model:

*Y_ij_ = B_0_+B_1_X_ij_+s_i_+b_i_X_ij_+e_ij_*,

Where, *Y_ij_* = dependent variable; *B_0_* = overall intercept across all studies (fixed effect); *B_1_* = linear regression coefficient of Y on X (fixed effect); *X_ij_* = tannin supplementation as the continuous predictor; *s_i_* = value of random effect of study *i*; *b_i_* = random effect of study on the regression coefficient of Y on X in study *i*; and *e_ij_* = the unexplained residual error.

## Results

All of the evaluated additions affected growth performance, lymphoid organ weight, and amino acid (AA) ileal digestibility, compared with the control group (Table-2). Broilers gained 42.12±27.68 g/bird/day, with an average daily FI (ADFI) of 76.14±50.66 g/bird/day, resulting in an FCR of 1.942±0.468.

**Table-2:** Summary of the descriptive statistics of the studies included.

Response parameters	Unit	Mean	SD	Max	Min
Performance					
Average daily gain	g/bird/day	42.12	27.68	92.71	6.56
Average daily feed intake	g/bird/day	76.14	50.66	189	10.73
Feed conversion ratio	no unit	1.942	0.468	3.48	0.97
Thigh	%	7.958	4.782	14.17	4.00
Drumstick	%	11.53	2.889	15.38	7.86
Wing	%	6.295	3.295	10.7	3.44
Fat	%	1.369	0.572	2.35	0.76
Liver	%	2.628	0.631	4.81	2.15
Lymphoid organ weight					
Bursa	%	0.689	0.92	3.2	0.22
Spleen	%	0.458	0.77	2.4	0.10
Amino acid ileal digestibility					
Lysine	%	87.8	1.643	89	85
Threonine	%	72.4	13.59	96	64
Isoleucine	%	82.2	2.683	85	78
Leucine	%	82	5.523	87	73
Phenylalanine	%	84.2	4.147	87	77
Arginine	%	80.2	6.221	87	70
Methionine	%	88.4	3.209	92	84

SD=Standard of deviation, Max=Maximum value, Min=Minimum value

The use of tannins as a feed additive had various effects on carcass parameters. Drumstick, thighs, and wings accounted for 7.96%, 11.53%, and 6.25% of thetotal carcass weight, respectively, followed by abdominal fatand liver parts at 1.37% and liver at 2.63%. Differences between the minimum and maximum values for carcass composition parameters were 3.54-fold for thighs, 1.96-fold for drumsticks, 3.13-fold for wings, 3.09-fold for fat, and 1.69-fold for the liver. Carcass composition was widely different for each broiler because of different strains and ages. In addition, the carcass composition and growth performance of broilers treated with tannin showed various results, as shown by the minimum and maximum values for each parameter. For the bursa and spleen, minimum and maximum values ranged from 0.22% to 3.2% and 0.1% to 2.4%, respectively. Conversely, tannins have little effect on digestibility. There was little variation in AA ileal digestibility wherethe largest difference in digestibility was threonine.

Table-3 presents the parameter estimates of the effect of dietary tannin levels on broiler performance, lymphoid organs, and AA digestibility. The present meta-analysis revealed that increasing dietary tannin levels had a negative effect on broiler ADG and ADFI, as shown by the linear decrease in the slopes (p<0.001; (Table-3); however, it did not affect FCR (p=0.47). In addition, increasing dietary tannin levels decreased drumstick and liver weight, as well as negatively affecting the bursa Fabricius and spleen weights (p<0.05). Meanwhile, other carcass traits (i.e., thighs, wings, and body fat) were not influenced by the inclusion of dietary tannins. In terms of AA digestibility, as the dietary concentration of tannins increased, it had a negative effect on isoleucine, leucine, and methionine digestibility, as shown by their negative slopes (Table-3). Other essential AA such as lysine, threonine, and arginine were not influenced by tannin levels.

**Table-3:** The effects of tannin supplementation levels on performance, lymphoid organ weight, carcass cuts weight, and amino acid ileal digestibility of broiler chickens.

Response parameters	Unit	Model	N	Intercept	SE intercept	Slope	SE slope	p-value	RMSE	AIC	Trend
Performance											
Average daily gain	g/bird/day	L	68	46.23	5.67	−4.87	1.13	<0.001	1.75	480.50	Negative
Average daily feed intake	g/bird/day	L	65	84.76	11.52	−9.62	1.97	<0.001	1.83	532.94	Negative
Feed conversion ratio	No unit	L	65	1.88	0.11	0.04	0.05	0.47	2.06	41.41	Positive
Thigh	%	L	16	7.60	3.18	−0.81	1.54	0.61	1.12	25.35	Negative
Drumstick	%	L	16	11.65	1.88	−6.96	1.93	0.004	1.11	28.62	Negative
Wing	%	L	16	6.02	2.20	0.09	1.22	0.94	0.99	18.19	Positive
Fat	%	L	10	1.34	0.44	−0.23	0.54	0.68	1.13	18.94	Negative
Liver	%	L	17	3.03	0.50	−0.66	0.21	0.010	1.01	26.15	Negative
Lymphoid organ weight											
Bursa	%	L	15	0.92	0.58	−0.28	0.09	0.010	1.30	25.76	Negative
Spleen	%	L	15	0.82	0.49	−0.62	0.09	<0.001	1.22	23.62	Negative
Amino acid ileal digestibility											
Lysine	%	L	5	89.52	1.48	−1.33	0.50	0.08	0.77	19.82	Negative
Threonine	%	L	5	64.42	19.69	6.14	6.69	0.43	0.77	35.36	Positive
Isoleucine	%	L	5	85.31	1.67	−2.40	0.57	0.02	0.77	20.54	Negative
Leucine	%	L	5	88.20	4.04	−4.77	1.37	0.04	0.77	25.85	Negative
Phenylalanine	%	L	5	88.37	4.06	−3.21	1.38	0.10	0.77	25.88	Negative
Arginine	%	L	5	86.79	5.45	−5.07	1.85	0.07	0.77	27.66	Negative
Methionine	%	L	5	92.21	1.70	−2.93	0.58	0.01	0.77	20.65	Negative

SD=Standard of deviation, Max=Maximum value, Min=Minimum value, N=Sample size; RMSE=Root means square error, AIC=Akaike information criterion

## Discussion

Among the entire dataset, the performance of broiler chickens was fairly good, as indicated by an ADG of 42.12±27.68 g/bird/day and an FCR of 1.94±0.47. This performance was nearly similar to that noted in the meta-analysis conducted by Létourneau-Montminy *et al*. [38]. The values for other variables such as carcass traits, lymphoid organs, and AA digestibility were also within the normal range [39].

In this meta-analysis, broilers had an average ADG of 42.12±27.68 g/bird/day, based on an average ADFI of 76.14±50.66 g/bird/day, resulting in an average FCR of 1.942. These results are comparable with the ADG, ADFI, and FCR of broiler chickens generally. Under normal conditions, at 35 days of age, Cobb and Ross strain 408 broilers have an ADG of 63.86 and 65.3 g/bird/day, respectively, based on an ADFI of 179 and 185 g/bird/day, respectively, leading to an FCR of 1.5 and 1.473. A similar variation was also noted for carcass components. Average values for thighs, drumsticks, and wings were 7.958%, 11.53%, and 6.25% of their total carcass weight, respectively, followed by fat and liver percentage values of 1.369% and 2.628%. Since the present study was comprised of multiple experiments, it is generally accepted that there were large variations among parameter estimates due to the difference in length of the experiments used. The high heterogeneity can be observed from the minimum and maximum values for both growth performance and carcass composition parameters, there was wide variation in the results (Table-2).

The implication from the high heterogeneity mentioned above is that the lymphoid organs were also in large variations (Table-2). Conversely, there was little variation in AA ileal digestibility, with the greatest effect noted in the digestibility of threonine. As the effect of the tannin on AA digestibility was only evaluated in one study with a low level of tannins (<0.5%) [34], this finding suggests that protein digestibility was not impaired by such levels. However, high doses of tannin may affect the development of primary and secondary lymphoid organs [14]; a compound within tannin causes a deficiency in protein utilization, leading to impaired immune function [40]. As such, the assumption of essential AA, minerals, and vitamins is decreased in the gut, affecting the growth of the lymphoid organs. Decreasing the lymphoid organ weight could lead to decreased the bursa Fabricius, thymus, and spleen weights [9].

Tannins are considered beneficial natural compounds for broiler chickens, particularly with respect to gut microbiota modulation; however, as additional evidence has been produced, inconsistent results have emerged from different studies. According to the literature, tannin effectively modulates gut microbiota and the immune system [14,41,42]; thus, it could potentially be used as a replacement for antibiotics. As such, researchers have expected tannin supplementation to have a growth-promoting effect. Conversely, the present meta-analysis confirmed contradictory results, in which elevating tannin levels almost completely suppressed growth performance, lymphoid organ weights, and AA digestibility. This detrimental effect was mainly due to the ability of tannins to form tannin-protein complexes in the intestine [43], hence reducing the availability of AA for digestion [44]. In addition, as an antinutritional factor, tannins appeared to decrease the secretion of digestive enzymes and their activity. Consequently, a portion of the nutrients cannot be utilized, particularly protein. The decreased AA digestibility may also be explained by a negative feedback mechanism in which undigested nutrients stimulate enzyme secretion, resulting in a decrease of indigenous AA [43]. In general, in broilers, tannin consumption at high doses decreases AA digestibility; a similar result was also reported by Avila *et al*. [45] and Mansoori and Acamovic [46], particularly for methionine, leucine, and isoleucine. Methionine, with a high affinity for AA [47], may directly reduce mucosal uptake because of a disturbance of the Na+ and K+ pump cotransportation of AAs or indirectly through the inhibition of Na+/K+ ATPase [46]. Excessive tannin concentrations decrease AA (mostly leucine and isoleucine) and support growth depression due to the secretion of enzymes by a hyperactive pancreas [48]; this effect would divert these AAs from the synthesis of body tissue protein to the synthesis of these enzymes, which are subsequently lost in the feces.

In the present meta-analysis, the deleterious effects of tannins were more obvious when tannins were supplemented at a higher level. Theprevious research has noted the negative effects of high dietary levels of tannins [24]. For instance, the weight gain and FCR of 42-day-old broiler chickens were impaired when they were fed a diet containing 0.56% and 0.28% tannins, respectively [43,49]; the authors also reported a decrease of aminopeptidase in chickens that were fed a high tannin content. Decreased broiler growth is likely a result of a reduction in AA digestibility but could also be due to a reduction of metabolizable energy [50]. The reduced bioavailability of protein and energy is two key factors that may explain the mechanism through which elevated tannin content decreases some broiler carcass traits [51], which was confirmed in this meta-analysis.

However, we must underline that dietary tannins may be beneficial when added at low levels, especially for antibacterial and immunostimulatory purposes. Over the past couple of years, a positive effect of the inclusion of low levels of tannins has been reported, particularly with respect to the ­stimulation of ­antioxidant status, improvement of intestinal morphology [52], positive modulation of intestinal microbiota [41], and improvement of mucosal immunity [53]. Regarding broiler performance, diets with low levels of tannins have been reported to have comparable effects as diets with no tannins. For example, a dietary concentration of 0.12% has no adverse effect on ileal AA digestibility [43]. Other studies have also reported no substantial effects of low levels of dietary tannins on broiler growth performance and carcass characteristics [43,44,54]. This meta-analysis indicates that dietary tannins can have some negative effects on broiler performance, lymphoid organ weight, and AA ileal digestibility.

## Conclusion

This meta-analysis suggests that increasing levels of dietary tannins impair the growth of broiler chickens, which reduces broiler performance; thus, we do not recommend including tannins in broiler diets.

## Authors’ Contributions

CH: Designed the study and collected the data. CH, AI, TRP, and YRY: Wrote original draft and reviewed final version of the manuscript. AJ and EW: Reviewed the final version of the manuscript. MMS, SS, RK, and II: Performed formal analysis and data curation. All authors read and approved the final manuscript.
